# Noninvasive Oxygen Monitoring in Three-Dimensional Tissue Cultures Under Static and Dynamic Culture Conditions

**DOI:** 10.1089/biores.2015.0004

**Published:** 2015-05-01

**Authors:** Birgit Weyand, Mariel Nöhre, Elmar Schmälzlin, Marvin Stolz, Meir Israelowitz, Christoph Gille, Herb P. von Schroeder, Kerstin Reimers, Peter M. Vogt

**Affiliations:** ^1^ Department of Plastic, Hand and Reconstructive Surgery, Hannover Medical School, Hannover, Germany.; ^2^ Colibri Photonics GmbH, Potsdam, Germany.; ^3^ Biomimetics Technologies, Inc., Toronto, Canada.; ^4^ University Hand Program and Bone Lab, Department of Surgery, University of Toronto, Toronto, Canada.

**Keywords:** cell culture, extracellular matrix, hypoxia, stem cells, tissue engineering

## Abstract

We present a new method for noninvasive real-time oxygen measurement inside three-dimensional tissue-engineered cell constructs in static and dynamic culture settings in a laminar flow bioreactor. The OPAL system (optical oxygen measurement system) determines the oxygen-dependent phosphorescence lifetime of spherical microprobes and uses a two-frequency phase-modulation technique, which fades out the interference of background fluorescence from the cell carrier and culture medium. Higher cell densities in the centrum of the scaffolds correlated with lower values of oxygen concentration obtained with the OPAL system. When scaffolds were placed in the bioreactor, higher oxygen values were measured compared to statically cultured scaffolds in a Petri dish, which were significantly different at day 1–3 of culture. This technique allows the use of signal-weak microprobes in biological environments and monitors the culture process inside a bioreactor.

## Introduction

Monitoring and delivering sufficient oxygen supply in three-dimensional (3D) tissue constructs pose a challenge for tissue engineering applications.^1,2^ The thickness and structure of the scaffold affect oxygen diffusion and nutrient supply, as well as removal of toxic waste, which is essential for the survival of cells seeded within the scaffold. Furthermore, after implantation of the scaffold into an organism, a rapid connection toward the capillary system is essential to ensure cell vitality.^3^

Whereas environmental oxygen concentration is 21%, the physiological oxygen levels in the blood range between 10% and 13%.^4^ Oxygen gas levels found in the terminal vascular bed in tissues are about 8% (5–10%), but show differences between highly vascularized tissues such as bone (∼5–10%) and bone marrow (∼2–7%) or avascular tissues such as cartilage (∼1–6%).^5–7^ Critical hypoxic levels <1% are found in sites of infection or necrosis. Strictly speaking, mM or mg/L is indicated as the unit for dissolved oxygen. However, it is common practice to use the unit % in physiological environments. Twenty-one percent means that the solution is air saturated, which corresponds to 6.7 mg/L at 37°C and 1013 hPa.^8^

The diffusion limit of oxygen in dense tissues, such as the skeletal muscle or bone, is less than 200 μm.^2^ This also poses a major problem for tissue-engineered cell constructs, since cells in the inner part of the scaffold are in danger of oxygen deprivation, which affects the cell metabolism, proliferation, and differentiation.^7,9^ Moreover, oxygen (dis)solubility in aqueous solutions such as the cell culture medium is quite limited compared to blood containing red blood cells with hemoglobin as a good oxygen carrier.

A certain level of physiological hypoxia is beneficial for cell growth and metabolism. Furthermore, atmospheric oxygen levels (21%) may even have deleterious effects on stem cells *in vitro*.^10^ The influence of physiological hypoxia (5–10%) on stem cell growth and differentiation is currently a significant research interest.^11–13^ On the other hand, an oxygen level at 1% or below can only be tolerated by the cells for a limited time, but can lead toward cell death when prolonged.^14^

The decreasing oxygen gradient inside a tissue-engineered scaffold is one reason for irregularly distributed cells often found in *in vitro* cultures with a dense cell population growing at the outside of the scaffold compared to the inner core lacking viable cells due to severe hypoxia. Other factors are structural properties of the scaffold such as porosity and interconnectivity that can adversely affect cell distribution. Cultivation of tissue constructs inside perfusion bioreactors has improved cell distribution and also enhanced cell survival by improving mass transport and oxygen supply.^9,15–17^ To determine the oxygen concentration in a tissue-engineered scaffold inside a bioreactor, two different approaches have been used so far: the oxygen concentration inside a scaffold is either calculated from indirect oxygen measurements at the inlet and outlet of a perfusion system^18,19^ or oxygen is directly measured through a micropipette, which has been inserted inside a scaffold.^7,9,20^ Both methods have limitations. The former method usually requires two oxygen sensors, whereas the latter method may affect the integrity of the scaffold or the micropipette itself.

Since oxygen levels correlate with cell numbers, cell growth, and survival, the monitoring of oxygen levels during bioreactor culture can monitor and also control the cultivation process.^9,14,16,20–23^ However, monitoring the culture process of a cell-seeded scaffold in a bioreactor during the experiment is limited. Direct observation tools are generally restricted to the surface layer of the tissue, where newer methods may reach down to about 200 μm depth.^24^ Indirect methods such as pH measurement, lactate concentration, or glucose consumption can give some additional information about the cell's well-being and growth.

In our current work, we describe a new method for noninvasive real-life monitoring of oxygen concentration in the center of a 3D scaffold inside a perfusion bioreactor. The method is based on a dual-frequency phase modulation technique for green light outside the reactor and phosphorescent microbeads, which are placed inside the scaffold. The technique has been first developed for the measurement of intracellular oxygen levels in green plants,^25–27^ and is now transferred to applications with mammalian cell culture.

## Materials and Methods

### Oxygen measurement system

**FIG. 1.**
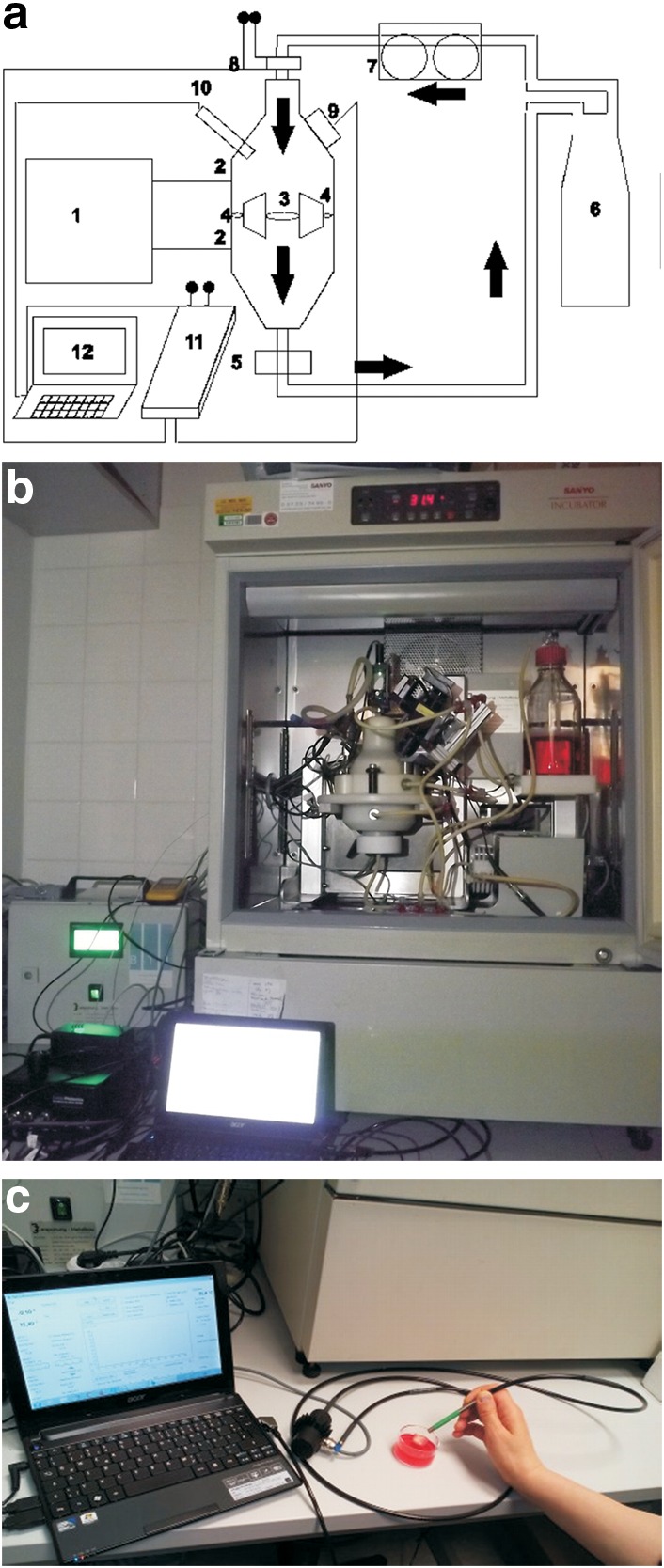
**(a, b)** Set up of system. **(a)** Schematics. 1, Control box; 2, pressure ports; 3, bioreactor vessel with scaffold holder; 4, integrated bypass system; 5, sampling probe (for medium analysis); 6, culture medium reservoir; 7, peristaltic pump; 8, laser light with filters and lenses; 9, photomultiplier (light detector); 10, temperature sensor; 11, OPAL system (optical oxygen measurement system) with built-in sinusoidal frequency generator and lock-in amplifier; 12, computer with software; **→** medium flow direction. **(b)** Bioreactor with laser. **(c)** Setup of static measurement system, the y-shaped light guide is connected to the light source, the light detector, and the OPAL system with the computer software.

For oxygen detection, we used an OPAL system (optical oxygen measurement system; Colibri Photonics), which was adapted to the bioreactor. The instrument measures the oxygen-dependent phosphorescence lifetime of spherical microprobes that are overgrown by the cell tissue under examination. A unique feature of the system is the use of a two-frequency phase-modulation technique,^25,26^ which fades out the interference of background fluorescence. This technique allows the use of signal-weak microprobes in biological environments. In previous studies, results obtained by the OPAL system for oxygen values in biofilms correlated strongly to reference data measured by a traditional Clark electrode.^28^ A description of the OPAL is given previously.^29,30^ The OPAL system consists of phosphorescent microbeads, a light-emitting diode (LED) or a laser light source, an electronic unit with an implemented lock-in-amplifier, a frequency generator and light source driver, a photomultiplier-based signal detector and software for data collection and analysis. The software is based on Microsoft Visual Basic 2010 (http://msdn.microsoft.com). Figure 1a shows the setup of the circuit, and Figure 1b shows the OPAL system connected to the bioreactor. For oxygen measurements of the static cell cultures, that is, cultures with scaffolds in Petri dishes, the LED source and the detector were linked to a Y-shaped light guide, which was used as a reflection probe, as shown in Figure 1c. For the use with the bioreactor, the LED source was replaced by a green 515 nm diode laser, which was fixed at the top of the reactor vessel.

### Cell culture

Primary human adipose mesenchymal stem cells were isolated from patients undergoing abdominoplasty after obtaining informed consent using a standardized protocol, as described elsewhere.^31,32^ The procedure was approved by the Ethics Committee of Hannover Medical School. Cells were cultured in the DMEM-F12 medium (PAA Laboratories) supplemented with 5% fetal calf serum, nonessential amino acids, sodium pyruvate (all Biochrom GmbH), penicillin–streptomycin–kanamycin (PAA Laboratories), and amphotericin at 37°C with 5% CO_2_. Multipotency of the isolated stem cells was routinely verified by differentiation into the adipogenic, chondrogenic, and osteogenic lineage and demonstration of common stem cell surface markers by flow cytometry (fluorescence-activated cell sorting analysis).^33–35^ Cells from passage 2 to 5 were used for experiments. The osteogenic differentiation medium was additionally supplemented with dexamethasone 10 nM, ascorbic acid 200 μM, and beta-glycerophosphate 10 mM.

### Scaffold preparation

Cylindrical scaffolds with a diameter of 10 mm and a height of 18–20 mm were cut out from a flexible bloc made from collagen–elastin fibers (MatriDerm^®^, Suwelack, kind gift from Prof. Dr. C. Kasper, BOKU, Vienna, Austria). Scaffolds were treated with isopropanol, air-dried under a laminar flow hood, and cut horizontally in two halves to access the center. Phosphorescent microbeads with 50-μm diameters (Pt-CPOx-Beads; Colibri Photonics GmbH) were suspended in 10–20 μL thrombin of a commercially available two-component fibrin glue (TISSEEL fibrin sealant; Baxter) and placed together with 10–20 μL of the fibrinogen component in the center half of the collagen cylinder. With further droplets of fibrin glue, the two halves of the collagen cylinders were brought together.

Two constructs were made. (1) Adipose mesenchymal stem cells were suspended in 300 μL of the culture medium and seeded inside the prepared scaffolds by injection; (2) 5% of the total number of stem cells intended for seeding was suspended in 10–20 μL of the fibrinogen component of the fibrin glue and placed together with the phosphorescent microbeads in the center of the scaffold, while 95% of the total number of stem cells was suspended in the culture medium and seeded by injection after composition of the scaffold.

Due to the spongy consistence of the collagen material, wet scaffolds had a height of about 14 mm with microbeads at a depth of about 5–7 mm, as measured from the top.

### Scaffold culture

Scaffolds were placed into glass Petri dishes with a diameter of 8 cm and a height of 3 cm and cultured for 24 h before the start of oxygen measurements in the DMEM-F12 medium with supplements in an incubator at 37°C with 20% O_2_ and 5% CO_2_. Two scenarios were tested. (1) Static two-dimensional (2D) cultures continued with the scaffolds in the Petri dishes in the incubator; the culture medium was changed thrice weekly. (2) For dynamic 3D cultivation, scaffolds were transferred to a bioreactor with addition of HEPES buffer solution (PAA-Laboratories) to the culture medium to maintain a stable pH and perfusion rate at 1 mL/min from top to bottom, as previously described.^17^ Pressure, temperature, and pH were continuously controlled at regular intervals.

### Bioreactor system

Based on a computational fluid model, the bioreactor design allowed laminar flow perfusion of scaffolds with pressure control through an integrated bypass system, as described elsewhere.^17,36–38^ The prototype bioreactor was adjusted for the optical noninvasive oxygen measurement system by a flow inlet with an optical window for the laser beam and a plexiglass insert and window on the vessel side for placement of the signal detector. The installation allowed noninvasive oxygen recordings of the centrally placed scaffold throughout the cultivation period at 37°C. Figure 1a and b show the system setup.

### Analysis

Cell proliferation was assessed by the CellTiter-Blue^®^ cell viability assay (Promega) according to the manufacturer's instructions (Promega) in 96-well plates. Apoptosis was determined by the Apo-ONE^®^ Homogeneous Caspase-3/7 Assay (Promega). Triplets/quadruplets of each time point were analyzed by spectroscopy at 560 nm emission and 590 nm excitation (GENios; Tecan). DNA was quantified by the NucleoSpin^®^ Tissue kit (Macherey–Nagel), following the manufacturer's instructions. DNA concentration was measured using NanoDrop (Thermo Fisher Scientific). For histology analysis, samples were fixed in formaldehyde solution, embedded in paraffin, and sectioned and stained with Alcian blue, Alizarin red, and von Kossa using standard protocols.^17^ Sections were analyzed under an inverse microscope (Olympus). Statistical analysis was performed by the single-factor analysis of variance and the two sample *t*-test (*p*<0.05) with Excel^®^ (Microsoft Office). Graphics show average values with standard deviation.

## Results

Before set up of the oxygen measurement system for the bioreactor system, the nontoxicity of the microbeads was verified by cell proliferation and lack of apoptosis through respective assays. Cells remained viable and continued to proliferate in cultures when in contact with microbeads, regardless of daily exposure to green excitation light (10–30 min) (Supplementary Fig. S1).

**FIG. 2.**
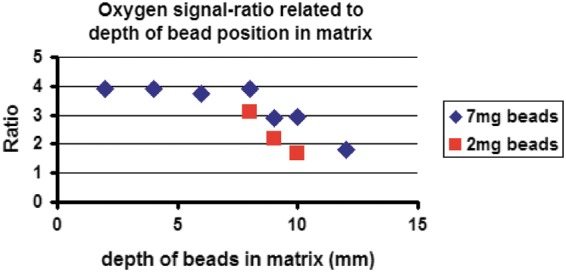
This graph demonstrates the relationship between the signal intensity and quality of the oxygen measurement system and the position and amount of phosphorescent microbeads in the collagen matrix. The x-axis shows the depth of the phosphorescent microbeads in the scaffold measured from top. The y-axis specifies the ratio of sensor phosphorescence to background fluorescence. A ratio >1:1 is sufficient for oxygen measurement with the OPAL system, which is <10°mm for 2 mg beads (red squares) and <12°mm for 7 mg beads (blue rhombus).

A stable signal of the phosphorescent microbeads was obtained inside a collagen scaffold up to 10–12 mm depth. Signal intensity and quality depended on penetration depth and the amount of microbeads shown in Figure 2, where the y-axis specifies the ratio of sensor phosphorescence to background fluorescence. With increasing depth in the matrix, the sensor phosphorescence fraction of the detected signal decreased. However, even at 12 mm depth, a ratio >1:1 was still achieved, which was sufficient for O_2_ measurements with the OPAL system.

**FIG. 3.**
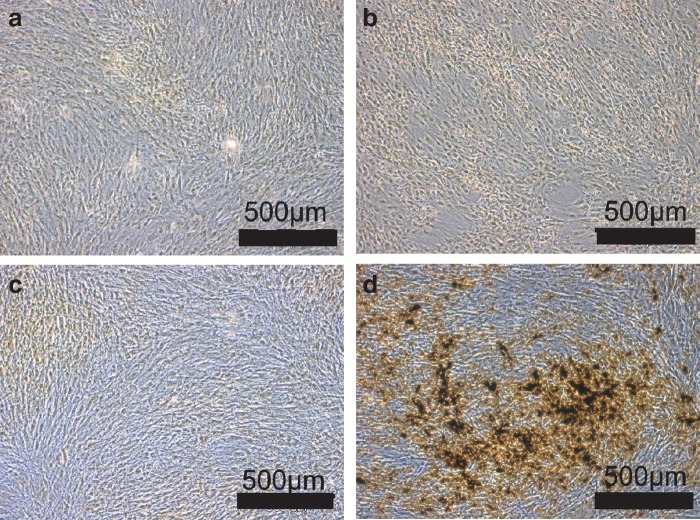
**(a–d)** Influence of oxygen tension on osteogenic differentiation of adipose mesenchymal stem cells (MSC) in static two-dimensional culture: MSC were cultivated in either standard stem cell media (control) or osteogenic (osteogenic) differentiation media for 4 weeks at 2% or 21% oxygen tension. At 2% and 21% oxygen tension, MSC cultured in the control medium showed no signs of osteogenic differentiation **(a, c)**. Hypoxic culture conditions inhibited osteogenic differentiation, despite use of the osteogenic medium, **(b)** whereas MSC cultured under normoxia in the osteogenic medium demonstrated pronounced osteogenic differentiation with extracellular matrix formation and typical mineralized deposits **(d)** (von Kossa staining). **(a)** Control 2% oxygen. **(b)** Osteogenic 2% oxygen. **(c)** Control 21% oxygen. **(d)** Osteogenic 21% oxygen.

Static cultures with mesenchymal stem cells were tested for the influence of normoxic (21%) and hypoxic (2%) culture conditions in either standard stem cell media or osteogenic differentiation media for 4 weeks. Hypoxic culture conditions inhibited osteogenic differentiation, whereas mesenchymal stem cells cultivated under normoxia demonstrated pronounced osteogenic differentiation with extracellular matrix formation and typical calcified deposits, as shown in Figure 3a–d (von Kossa staining).

**FIG. 4.**
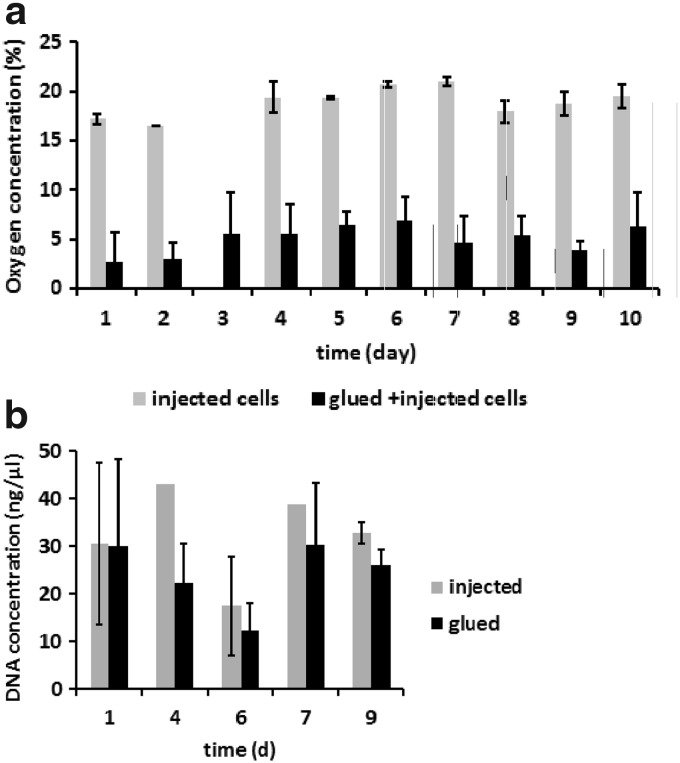
**(a)** Comparison between the two different cell–scaffold constructs tested in this study with (1) solely injected (gray) and (2) injected and glued cells (black) in the scaffold. Oxygen concentration depends on cell densities; when part (6×10^5^) of the total cell count was centrally glued, a significantly lower oxygen tension was measured in the center of the scaffold (*p*<0.05 at day 1 and 4–10 in static culture). **(b)** Comparison of DNA analysis of statically cultured scaffolds with injected (gray) or glued cells (black) (0.5×10^6^ stem cells/collagen disc) showed no significant differences between the two groups.

Oxygen concentration correlated with cell densities inside the scaffold. Scaffolds with randomly injected cells (1.5×10^7^ cells) showed oxygen concentrations in the center at 16–21%. In contrast, significantly lower oxygen concentrations at 2–6% were measured by the sensor system within the center of the scaffold constructs that contained both central cells (6×10^5^) plus randomly injected cells (1.3×10^7^ cells). Figure 4a demonstrates the time course of oxygen values in the two groups under static culture conditions. Statistically significant differences were found at day 1 and day 4–10.

The influence of fibrin glue on cell performance and survival was tested by DNA analysis. To do this, collagen discs were seeded with 0.5×10^6^ stem cells, either by injection or by fibrin glue, cultured statically for 7–9 days, and DNA analysis was done at various time points. There was no significant difference in the DNA concentration found between the two groups and between different time points (Fig. 4b).

**FIG. 5.**
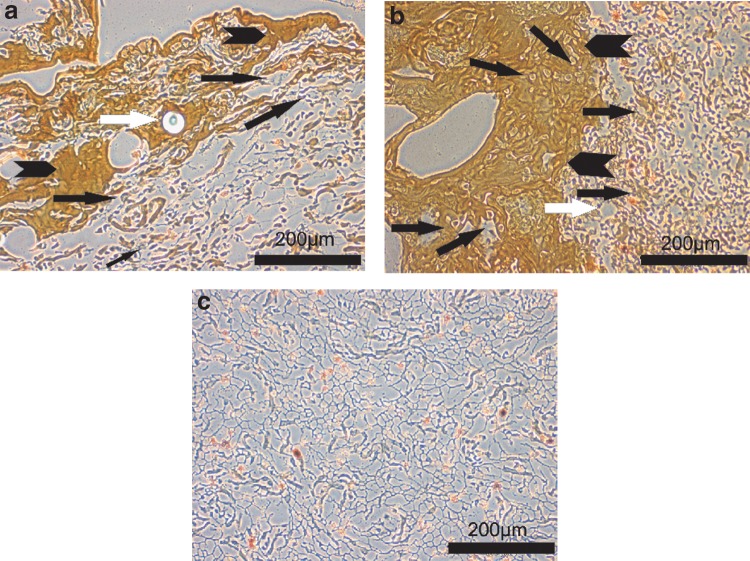
**(a–c)** Comparison of cell densities seen centrally in collagen scaffolds with **(a)** solely injected and **(b)** injected and glued cells. **(c)** Gives an example of the collagen matrix structure without seeded cells. **(a)** The histology of a statically cultured collagen scaffold with injected cells (black arrow), fibrin glue (black arrowhead), which stains brownish, and glued microbeads (white arrow) shows a low cell density in the central part. **(b)** The histology section of a statically cultured collagen scaffold with glued and injected cells (black arrows), fibrin glue (black arrowhead), and microbead (white arrow shows space where bead had been located) demonstrates a higher cell density compared to **(a)**. **(c)** A histology section of the plain collagen matrix structure without cells is shown for comparison.

Histology findings of static cultures revealed nonuniform cell clusters in scaffolds, where cells were only injected (Fig. 5a) compared to dense cell clusters embedded partly in fibrin in scaffolds, where cells were both glued and injected centrally (Fig. 5b). Figure 5c shows the structure of the pure collagen matrix without cells.

**FIG. 6.**
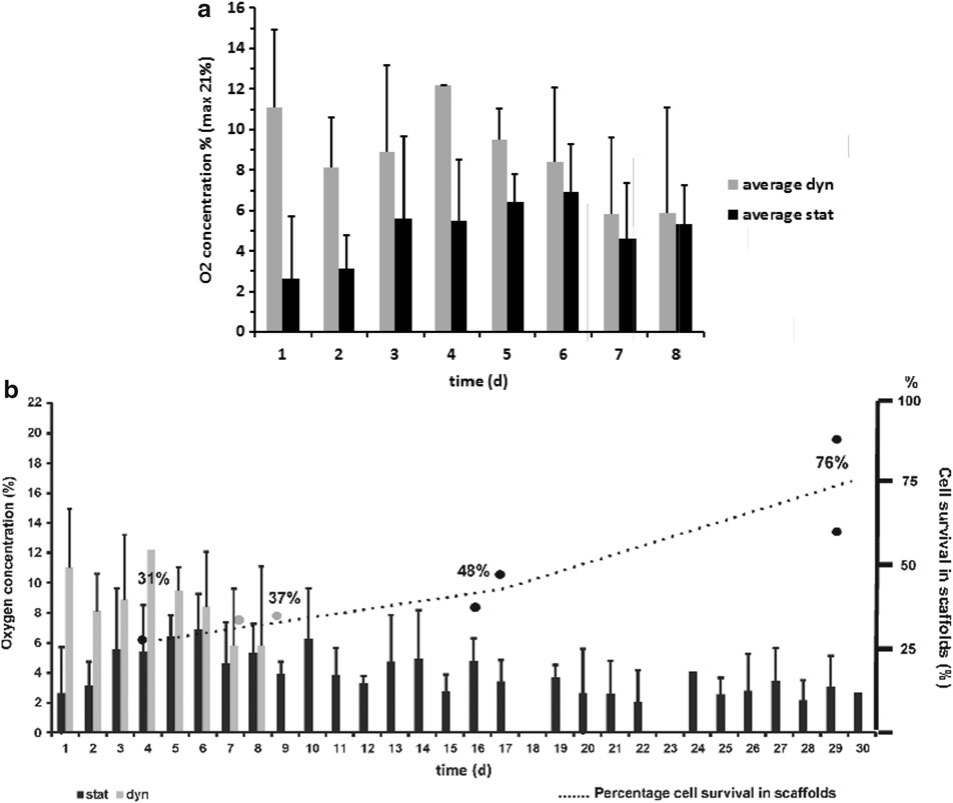
**(a)** Comparison of oxygen values measured in static and dynamic cultures. During the first 3 days, oxygen concentration was significantly higher in the bioreactor cultures (gray) compared to the static cultures (black). **(b)** Low oxygen concentrations as found in long-term static cultures (black) were accompanied by a slow steady increase in the DNA amount implicating cell proliferation. Oxygen concentration measured in static and dynamic cultures was plotted together with percentages of DNA content normalized to initially seeded cell numbers against the time. Black bars indicate the oxygen concentration measured in static cultures, which was in the range between 2% and 7% oxygen content, whereas gray bars stand for the values obtained in dynamic cultures (here, restricted to the first 8 days of culture, where scaffold integrity was fully preserved as discussed above), which were found between 6% and 12% oxygen content. The percentage scale on the right side of the plot shows the percentage of cell survival in the whole scaffold at different time points, which were calculated from DNA content obtained at the individual time point, cell numbers used for seeding the scaffold, and average seeding efficiency of the experimental setup. At day 4, cell counts calculated from DNA concentration were about 30% of the initially seeded cell numbers (black filled circle), which rose up to 76% of the initially seeded cell numbers during a 4-week time course in static culture. Values for bioreactor cultures (gray circles) showed comparable numbers at 1 week of culture with about 37% **(b,** in gray**)**.

During the first days of cultivation, values for oxygen concentration measured inside the scaffolds were higher in the dynamic culture compared to the static culture. Oxygen concentration in the center of the scaffold, cultured in the bioreactor, was found to be in the range of 8–12% in the first 6 days, whereas oxygen values measured in the static culture were in the range of 2–6%, as shown in Figure 6a. The differences were found to be statistically significant at day 1–3.

Low oxygen concentrations as found in long-term static cultures were accompanied by a slow steady increase in the total DNA amount of the scaffold, indicating cell proliferation (Fig. 6b). In Figure 6b, the oxygen concentration measured in static and dynamic cultures is plotted together with percentages of DNA content normalized to initially seeded cell numbers against the time. Black bars indicate the oxygen concentration measured in static cultures, which was in the range between 2% and 7% oxygen content, whereas gray bars stand for the values obtained in dynamic cultures (here, restricted to the first 8 days of culture, where scaffold integrity was fully preserved as discussed below), which were found between 6% and 12% oxygen content. The percentage scale on the right side of the plot shows the percentage of cell survival in the whole scaffold at different time points, which were calculated from DNA content obtained at the individual time point, cell numbers used for seeding the scaffold, and average seeding efficiency of the experimental setup. At day 4, cell counts calculated from DNA concentration were about 30% of the initially seeded cell numbers (black filled circle), which rose up to 76% of the initially seeded cell numbers during a 4-week time course in static culture. Values for bioreactor cultures (gray circles) showed comparable numbers at 1 week of culture with about 37% (Fig. 6b, in gray).

**FIG. 7.**
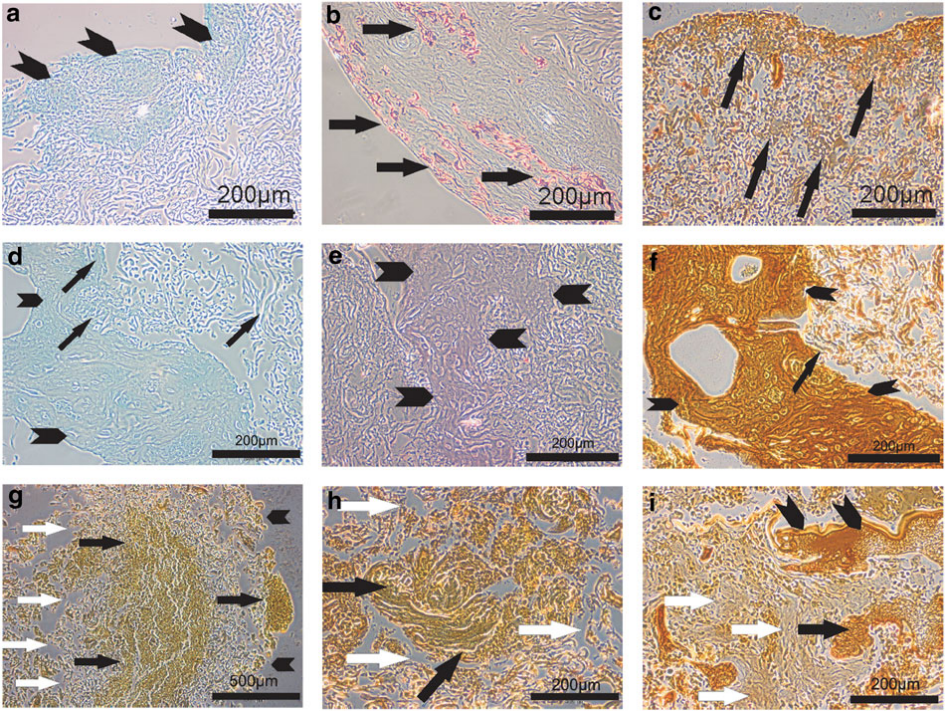
**(a–c)** Histological evaluation demonstrated positive markers for bone differentiation in long-term cultures. Deposits for glycosaminoglycans (Alcian blue staining), sparsely calcium deposits (Alizarin red staining), and several mineral deposits (von Kossa staining) were observed mainly toward the rand layer in 4-week static cultures. **(a)** The rand layer of a 4-week statically cultured collagen scaffold shows blue-stained glycosaminoglycan deposits (black arrowhead) (Alcian blue stain). **(b)** Red-stained calcium deposits (black arrows) are found in the rand layer of a statically cultured collagen scaffold at 4 weeks (Alizarin red staining). **(c)** Newly formed extracellular matrix with mineral deposits (black arrows) is stained with von Kossa staining in the rand layer of a statically cultured collagen scaffold at 4 weeks. **(d–f)** In the centrum of statically cultured scaffolds, less signs of bone differentiation markers are observed at 4 weeks, as shown in (**d–f)**. **(d)** The fibrin clot at the centrum of a statically cultured scaffold (arrowheads) is bordered on very small glycosaminoglycan deposits (black arrows) inside the collagen scaffold (Alcian blue staining). **(e)** Alizarin red staining shows no signs for red calcium deposits in the collagen scaffold around the centrally placed fibrin clot (arrowheads) (Alizarin red staining). **(f)** The centrally placed fibrin clot shows a brownish ground staining with von Kossa (arrowheads), while there are only very few dark, stained mineralized deposits (black arrow) in between the surrounding collagen matrix in statically cultured scaffolds (von Kossa staining). **(g–i)** Histology of dynamically cultured scaffolds in bioreactor (von Kossa staining). **(g)** The section of a 4-week dynamically cultured scaffold shows mineral deposits centrally and peripherally (black arrows) and disintegration of the collagen matrix (white arrows). The edge of the remaining scaffold is marked by black arrowheads. **(h)** A higher magnification of a dynamically cultured scaffold is shown with a centrally located mineralized matrix (black arrows) and disintegration of the collagen matrix (white arrows) (von Kossa staining). **(i)** At 4 weeks of culture, part of the fibrin (black arrowheads) with some embedded cells (black arrow) is still seen centrally in the dynamically cultured scaffold. Adjacent regions with new extracellular matrix formation (white arrows) can be observed.

Histological evaluation showed positive markers for bone differentiation in long-term dynamic 3D cultures. Despite a complete absence of any osteogenic differentiation stimuli, such as culture media supplements or external forces, static cultures demonstrated some deposits of glycosaminoglycans, sparse calcium deposits, and mineral deposits, mainly in the edge of the scaffold after a culture period of 4 weeks (Fig. 7a–c). In the center of statically cultured scaffolds, fewer signs for bone differentiation markers were observed at 4 weeks, as shown in Figure 7d–f. In scaffolds that were dynamically cultured in the bioreactor for 4 weeks, the collagen matrix of the scaffolds started to disintegrate, whereas in the remaining parts, we observed mineral deposits and extracellular matrix formation that were found more densely distributed at 4 weeks (Fig. 7g–i). Interestingly, some of the fibrin glue in the center was still found at 4 weeks of culture, partly also with embedded cells.

## Discussion

To measure and monitor oxygen concentrations, various optical methods by spectroscopic or adsorptiometric sensing and luminescent quenching with phosphorescent probes have been described in the past.^39–42^ The optical methods are based on phosphorescence quenching. To determine the oxygen-dependent phosphorescence decay time of an optical oxygen sensor, it can be excited by a sinusoidal modulated light. Dependent on the ambient oxygen concentration, the phosphorescence signal of the sensor is time delayed, resulting in a phase shift between excitation and phosphorescence light. Measuring the phase shift allows evaluation of the lifetime of the excited state of the sensor molecule and, in turn, the calculation of the related ambient oxygen concentration through a calibration curve. Our two-frequency phase-modulation technique is able to overcome the strong interference by background fluorescence signals arising from the collagen matrix, the culture medium, and the cell tissue; modulating the excitation light with two superposed sinus frequencies and measuring the respective phase shifts allow to separate fluorescence and phosphorescence fractions and to recalculate the actual oxygen-dependent sensor lifetime.^25^ This technique enables the use of signal-weak microprobes and the remote detection of their signal through the cell carrier and the culture medium.

During the establishment of our measurement setup for the bioreactor, first experiments on mesenchymal stem cells were done with blue excitation light. In this study, an increased rate on death cells in the life/dead assay was observed when cells were exposed to blue light for 30 min daily, independent of the presence or absence of phosphorescent microbeads. Long-term exposure to blue light has been associated with changes in mitochondrial function and induction of cell death in cell lines.^43^ Furthermore, some ingredients in cell culture media such as riboflavin can generate free oxygen radicals after light exposure and therefore an optical excitation of these ingredients needed to be excluded for our experimental setup.^44^ To our knowledge, there have been no reported adverse effects of green light exposure to cultured cells. In our experimental setup, the daily measurement period consisted of less than a 5 min light exposure of the cultured stem cells; hence, any adverse effects on cell culture, such as increased heat emission by the excitation laser, could be negated, and we did not observe any adverse effects on cell proliferation and survival by the green excitation light.

Our oxygen sensor system was suitable to detect cell densities and monitor cell proliferation during an experimental time course; a significant decrease in oxygen content measured in the centrum of a scaffold was detected. Higher cell densities in proximity to the fixed microbeads, which were achieved by adding cells into the fibrin glue compared to cells merely injected in the scaffold, were associated with a lower oxygen concentration measured by the sensor. In long-term static culture, a steady increase in DNA content from day 4 onward was accompanied by a decrease in oxygen values. Since the DNA content is determined for the whole scaffold, whereas the oxygen concentration is measured more punctually in the center of the scaffold, this correlation has to be interpretated with caution. However, for calculation of cell numbers, the method would require a three-point measurement and the scaffold would need a different internal structure with higher porous interconnectivity to allow better cell migration and more homogenous cell distribution. Commercially available MatriDerm^®^ is supplied in thin sheets with a thickness of 0.5, 1, and 2 mm and usually does not demonstrate the problems experienced with our thick blocs of MatriDerm collagen sponge (dry thickness of 18 mm, wet thickness about 15 mm) used in this study, for example, in respect to the low cell-seeding efficiency seen in our experimental setup.

Human mesenchymal stem cells in static 2D cultures showed inhibited osteogenic differentiation when cultured under reduced oxygen tension (2%). Similar observations have been observed by others in the past.^5,11,13,45–48^ However, in our dynamic 3D culture system in the collagen scaffold, signs of osteogenic differentiation were found after 4 weeks of static and dynamic culture, despite the lack of osteogenic supplements in the culture medium. In this study, the 3D structure itself together with the matrix material composition of collagen, which is the major structural component of the human musculoskeletal system, might be the main stimulus for spontaneous osteogenic differentiation of the mesenchymal stem cell population. In long-term static cultures, the central part of the scaffold where low oxygen values had been measured, showed less mineralization, whereas some calcified deposits were seen toward the outer layers of the scaffold, where higher oxygen concentrations are to be expected. Continuous dynamic culture with flow shear stresses led to new matrix formation and ossification inside the scaffold. Shear stresses have been already recognized as an important stimulating factor of osteogenic differentiation in recent studies.^49–52^ In the present study, despite the pronounced osteogenic differentiation found in dynamically cultured scaffolds, we observed disintegration and instability of the structural components of the collagen matrix under long-term dynamic perfusion culture. Therefore, cultivation time had to be shortened and values of oxygen measurements were only taken during the first week of dynamic culture when the scaffold showed a reliable structural integrity.

Monitoring metabolic processes or oxygen concentration inside a scaffold during culturing in a bioreactor is challenging. A noninvasive imaging method using two-photon excited fluorescence has been applied in a perfusion bioreactor to continuously monitor metabolic changes inside a porous silk scaffold with a coculture of adipose stem cells and endothelial cells up to a depth of 200 μm.^24^ By monitoring the redox ratio of the scaffold, a change in the cell redox ratio between the first and second week of dynamic culture was determined to indicate the beginning of differentiation of adipose stem cells.^24^

Regarding oxygen sensing, some experiences have been reported with a sensor in the form of a microneedle (PreSens), either with a preformed tunnel inside the demineralized bone scaffold at a depth of 2.5 mm ^7^ or a tantalum scaffold at a depth of 5 mm ^20^ or through a guide silk tubing inside a collagen gel^53^ by means of a micromanipulator. This procedure is elaborate and delicate, but allows the stepwise monitoring of oxygen gradients from the edge toward the center of a scaffold. The measurement results correlated with cell viability, with oxygen levels at 0%, went along with cell death.^7^

Another method for monitoring oxygen concentration in perfusion bioreactors is the measurement of dissolved oxygen tension either at the flow inlet and outlet^22,54^ or only at the flow outlet with controlled oxygen tension at the inlet.^16^ This technique allows a global estimate of cell numbers in the scaffolds, being able to determine even low cell numbers down to 10^5^ cells.^16^ Some errors may occur due to suspended cells in the perfusion chamber that have not been attached to the scaffolds, but are taken into account in the changes in oxygen values.

In summary, we have developed a noninvasive sensor system that allows monitoring of oxygen concentration inside tissue-engineered scaffolds when cultured statically or dynamically in a bioreactor. The method allows elimination of autofluorescence and works without disruption of the scaffold itself during the measurement process, but does require controlled positioning and fixation of phosphorescent microbeads inside the scaffold structure and a stable matrix during the experimental time course. Further studies are required to introduce this method to different biomaterials, which are better suitable for long-term culture in the bioreactor, to obtain detailed information about the influence of oxygen tension inside a tissue-engineered construct on cell proliferation and differentiation.

## Supplementary Material

Supplemental data

## Abbreviations Used

2D: two dimensional
3D: three dimensional
LED: light emitting diode
MSC: mesenchymal stem cells
OPAL: optical oxygen measurement system
