# Supplementary material for: Noninvasive Oxygen Monitoring in Three-Dimensional Tissue Cultures Under Static and Dynamic Culture Conditions
Source: Biores Open Access. 2015 May 1;4(1):266–77. doi: 10.1089/biores.2015.0004 (PMC4497672; doi:10.1089/biores.2015.0004)
Supplement: Supplemental data [file Supp_Figure1.pdf]

Supplementary Data

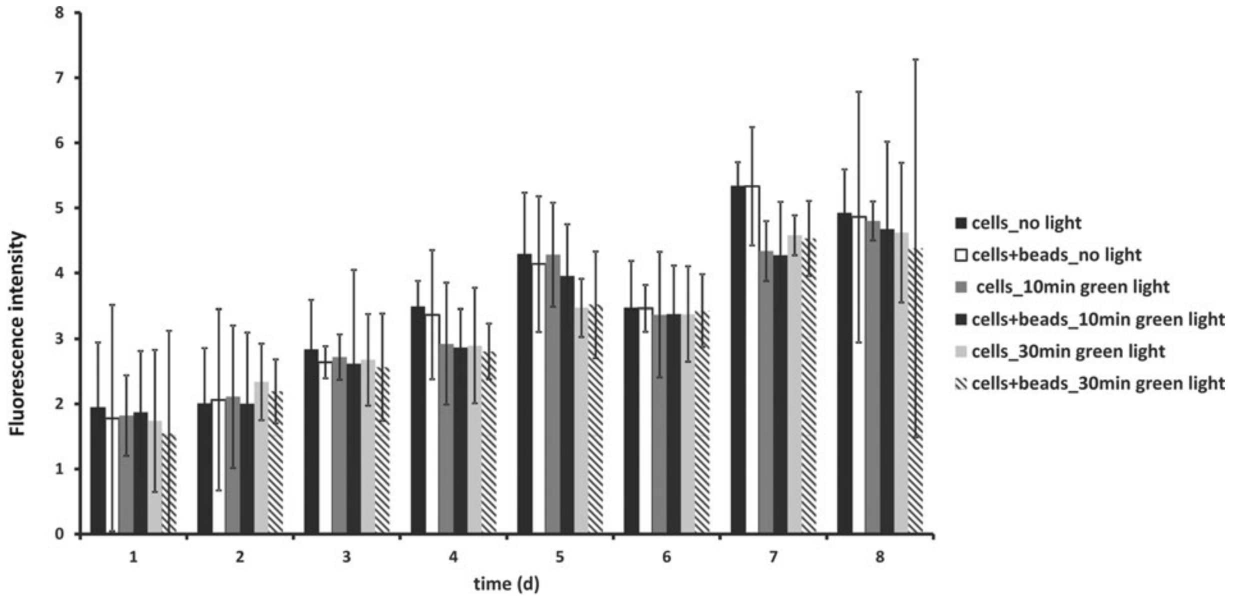

SUPPLEMENTARY FIG. S1. This figure shows the proliferation curves of mesenchymal stem cells grown in presence and absence of microbeads with and without exposure to green light for 0, 10 and 30 min, respectively. No significant differences were found between the different groups compared to controls.
